# Molecular mechanisms underlying gallic acid effects against cardiovascular diseases: An update review

**Published:** 2020

**Authors:** Ghaidafeh Akbari

**Affiliations:** 1 *Medicinal Plants Research Center, Yasuj University of Medical Sciences, Yasuj, Iran*

**Keywords:** Gallic acid, Cardiovascular diseases, Molecular mechanisms

## Abstract

**Objective:**

The prevalence of cardiovascular diseases (CVDs) is growing. CVDs are the major cause of mortality and have become one of the most important health challenges in developing countries. Gallic acid (GA) is a natural phytochemical which has been widely used against multiple conditions. The present review was designed to evaluate molecular mechanisms underlying the protective effects of this agent against CVDs.

**Materials and Methods:**

Data discussed in this review were collected from the articles published in databases such as Science Direct, Scopus, PubMed, and Scientific Information Database between 1993 and 2018.

**Results:**

According to the experimental studies, GA has protective actions against CVDs through increasing antioxidant enzymes capacity, inhibition of lipid peroxidation and decreasing serum levels of cardiac marker enzymes, modulation of hemodynamic parameters, recovery of electrocardiogram aberrations, and preservation of histopathological changes.

**Conclusion:**

GA has potential cardioprotective action. Therefore, it has been suggested that this agent can be administered in underlying of CVDS.

## Introduction

 Cardiovascular diseases (CVDs) are the first etiology of death all over the world. Different forms of CVDs are atherosclerosis, coronary artery disease, arrhythmia, and heart failure (Kang et al., 2015 ▶). Risk factors which contribute to CVDs are either unmodifiable (e.g. family history, race and age) or modifiable (e.g. hypertension, high cholesterol, obesity, type 2 diabetes). Thus, primary prevention of CVDs by determining and treating at-risk individuals remains a major public-health problem (Stone et al., 2014 ▶). Antioxidant enzymes including superoxide dismutase (SOD), catalase (CAT), glutathione peroxidase (GPx), glutathione reductase (GRx) and glutathione-s-transferase (GST) are the major cellular defense against injury (Priscilla and Prince, 2009 ▶). In the heart, glutathione (GSH) is very important because it can remove organic and inorganic peroxide (Kaul et al., 1993 ▶).

 It was reported that administration of fresh fruits, vegetables or plants rich in antioxidants, can lead to prevention of CVDs. Because low cost and less adverse effects, the use of traditional medicine is preferred over chemical drugs (Kumar et al., 2012 ▶). The protective effects of these natural products can be attributed to the presence of ﬂavonoids, anthocyanins, and other phenolic compounds (Zhang and Wang, 2002 ▶). In this regard, GA due to free radical scavenger and antioxidant action, received much attention (Stanely Mainzen Prince et al., 2009 ▶).

**Figure 1:**
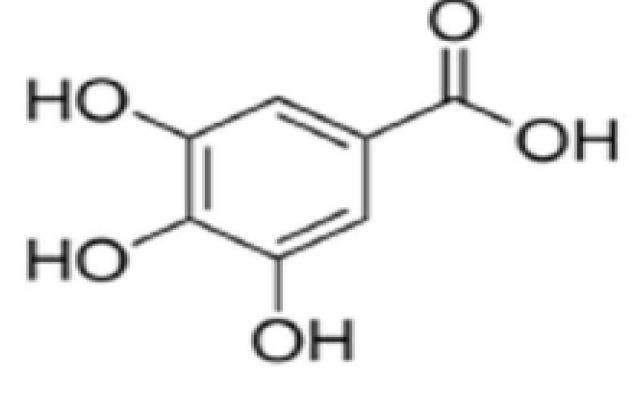
Chemical structure of gallic acid (3, 4, 5‑trihydroxy benzoic acid)

GA (Figure 1) belongs to the larger group of plant polyphenols known as gallotannins. It is found in vegetables, fruits (Kawada et al., 2001 ▶), tea leaves, grapes, blackberry, and gallnuts (Choubey et al., 2015 ▶). It has multiple biological effects such as antioxidant (Soong and Barlow, 2006 ▶), antiallergic (Kim et al., 2005 ▶) antimicrobial (Chanwitheesuk et al., 2007 ▶), anticancer (Wang et al., 2014 ▶), antiulcer (Sen et al., 2013 ▶), and neuroprotective properties (Mansouri et al., 2013 ▶)**. **

Furthermore, it has shown beneficial effects in animal model of metabolic diseases (Doan et al., 2015 ▶). In addition, GA has antihyperglycemic (Huang et al., 2016 ▶), and lipid homeostasis (Chao et al., 2014 ▶) actions.

While considerable evidence support that GA is a cardiprotective agent (Appeldoorn et al., 2005 ▶, Kee et al., 2014 ▶, Jin et al., 2017 ▶), no review article in this regard was published. Therefore, the aim of this review is to dicsuss the molecular mechanisms underlying GA effect against CVDs as summerized in Tables 1 and 2.

**Table 1:** Effect of different doses of gallic acid on CVDs

**Reference**	**Intervention/Duration/Animal/Route**	**Study design**
(Ryu et al., 2016 ▶)	100 mg/kg/1 week pretreatment and 2 weeks co-administration with ISP/mice/ip	Isoproterenol-induced cardiac hypertrophy and fibrosis
(Kulkarni and Swamy, 2015 ▶)	15 and 30 mg/kg/ 2 weeks/rat/po	Doxorubicin‑induced myocardial toxicity
(Dianat et al., 2013 ▶)	10, 30, 50 mg/kg/10 days/rat/gavage	CaCl_2_-induced arrhythmia
)Jinet al., 2017(	320 mg/kg/16 weeks/rat/po	Cardiac hypertrophy and spontaneously hypertension
(Badavi et al., 2014 ▶)	7.5,15,30 mg/kg/10 days/rat/gavage	Cardiac IR injury
)Ramezani-Aliakbari et al., 2017 ▶)	25 mg/kg/8 weeks/rat/gavage	IR following alloxan-induced diabetes
(Stanely Mainzen Prince et al., 2009 ▶)	15 mg/kg/10 days/rat/po	Isoproterenol-induced myocardial infarction
(Patel and Goyal, 2011 ▶)	100 mg/kg/8 weeks/rat/p.o 25,50,	Diabetes-induced myocardial dysfunction
(Umadevi et al., 2014 ▶)	25 mg/kg/30 days/rat/gavage	Advanced glycation end Products- induced cardiac remodeling
(Jin et al., 2017 ▶(	50,100 mg/kg/3 weeks/mice/ip 100 mg/kg/ip	L-NAME-induced hypertension Cardiac remodeling, and fibrosis
(Rather and Saravanan, 2013 ▶)	10 mg/kg/3 weeks/rat/ip	Immobilization-induced stress in myocardium
(Badavi et al., 2016 ▶)	30 mg/kg/28 days/rat/ gavage	Cirrhosis-induced ECG changes
(Badavi et al., 2017 ▶)	mg/kg/6 weeks/rat/ gavage and 40 20	Diabetes-induced MVB dysfunction

## Materials and Methods

Online databases including Science Direct, Scopus, PubMed, and Scientific Information Database between 1993 and 2018 using the keywords Gallic acid, Cardiovascular diseases and Molecular mechanisms.

## Results

**The effect of gallic acid on myocardial infarction**

Myocardial infarction (MI) is one of the major causes of death among CVDs which occur when myocardial oxygen demand is higher than oxygen supply and eventually leads to cardiomyocyte necrosis (Stanely Mainzen Prince et al., 2009 ▶). MI affects mechanical, electrical, structural and biochemical properties of the heart (Bakheet et al., 2014 ▶). 

A method for diagnosis of cardiac injury is measurement of the cardiac marker enzymes such as aspartate transaminase (AST), alanine transaminase (ALT), creatine kinase (CK), creatine kinase-MB (CK-MB), lactate dehydrogenase (LDH), and cardiac troponin T (cTnT) in serum. Among these, cTnT is a very sensitive, and speciﬁc parameter in detecting MI (Janota, 2014 ▶). 

It was reported that pretreatment with GA decreased the levels of these enzymes in serum probably due to reduction of myocardial damage and thereby limiting the leakage of these enzymes from myocardium (Priscilla and Prince, 2009 ▶). GA may protect the heart by inhibiting lipid peroxidation because it scavenges the superoxide, and hydroxyl radicals (Jadon et al., 2007 ▶). In addition, GA inhibits the lysosomal membrane destruction following isoproterenol-induced heart damage, and recovered the functions of this organelle to near normal levels. This activity of GA was attributed to antilipoperoxidative, and antioxidant characteristics of this agent (Stanely Mainzen Prince et al., 2009 ▶).

**Effect of gallic acid on vascular diseases**

The normal endothelium of vessel regulates tone, and structure, and exerts anticoagulant, and antiplatelet properties (Sandoo et al., 2010 ▶). The maintenance of vascular tone is done by the release of multiple vasodilator, and vasoconstrictor agents. The most important vasodilators are nitric oxide (NO), endothelium-derived relaxing factor (EDRF), prostacyclin, and bradykinin. The endothelium also produces vasoconstrictor substances, such as endothelin and angiotensin II (Bakheet et al., 2014 ▶). In hypertension (HTN), the balance between vasodilators and vasoconstrictors is disrupted (Nadar et al., 2004 ▶).

HTN is a public problem all over the world (Jin., et al. 2017 ▶), and is regarded as a major cardiovascular risk factor which leads to atherosclerosis, cardiac hypertrophy, and heart failure (Jin et al., 2017 ▶). It is a major cause of the occurrence of CVDs and left ventricular hypertrophy (LVH) (Verdecchia et al., 2004 ▶). Other pathophysiologic events of HTN are activation of the renin-angiotensin-aldosterone system (RAAS), endothelial dysfunction, salt consumption, and oxidative stress (Oparil et al., 2003 ▶). 

 In HTN, oxidative stress promotes vascular remodeling, as well as fibrosis, and hypertrophy (Harvey et al., 2016 ▶). Free radical oxygen species influence on nicotinamide adenine dinucleotide phosphate (NADPH) oxidase (Nox) (Jin et al., 2017 ▶). Nox2 is activated by angiotensin II or endothelin-1 (Sag et al., 2014 ▶). Nox2 has a more important role compared to the other Noxs in mediating oxidative stress response in cardiomyocytes. It has been reported that GA attenuated cardiac Nox2 transcript level, and Nox2 protein expression in spontaneously hypertensive rats (SHRs) (Jin et al., 2017 ▶).

 GA also down-regulates two constituents of RAAS including the angiotensin II receptor and angiotensin II-converting enzyme. Besides, GA decreased AT1 mRNA levels in the aorta, heart, and kidney cortex of SHRs but enhanced ACE1 mRNA levels in SHR aortas (Jin et al., 2017 ▶). GA also decreased HTN via a vasorelaxant effect by increased NO levels following activating phosphorylation of endothelial nitric oxide synthase (eNOS) (Kang et al., 2015 ▶). 

**Figure 2:**
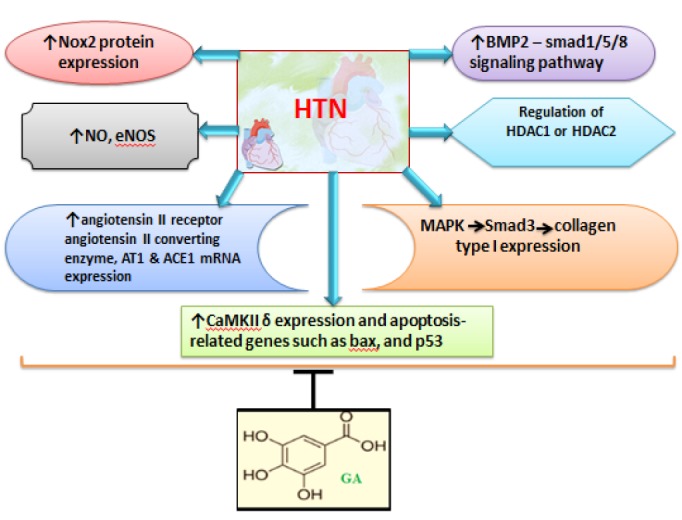
Schematic presentation of the molecular mechanisms underlying GA effects on HTN

Furthermore, GA down-regulates Ca^2+^/calmodulin-dependent protein kinase II δ (CaMKII δ) expression and apoptosis-related genes such as Bcl-2- associated X protein (BAX), and p53 mRNA levels in SHR (Jin et al., 2017 ▶). GA also decreases vascular calcification through the bone morphogenetic proteins (BMP2)–small mother against decapentaplegic (smad)1/5/8 signaling pathway inhibition, suggesting that GA may have a protective role in vascular diseases (Kee et al., 2014 ▶). as summarized in Figure 2.

**Effect of gallic acid on ECG abnormalities**

Arrhythmias determined by irregularlies at impulse production, impulse conduction or composition of both (Dianat et al., 2013 ▶). The most important etiologies of cardiac arrhythmias are MI, valvular heart problems, electrolyte disturbances, metabolic syndrome, and drug overdose (Zern and Fernandez, 2005 ▶).

 Ventricular fibrillation (VF) and ventricular tachycardia (VT) are two major etiologies of mortality in developing countries (Balasundaram et al., 2013 ▶). VF is a serious arrhythmia which is manifested by undistinguished rhythm, whereas, VT is a regular arrhythmias, and occur when the frequency of premature ventricular beat (PVB) becomes >3-4 beats/min (John et al., 2012 ▶). 

 Reperfusion-induced arrhythmias such as VT, VF, and PVB are the most important causes of cardiac death which happen due to excessing production of ROS, and calcium during first time of reperfusion (Zhao et al., 2010 ▶). It has been shown that GA decreases the incidence of VF, VT, and PVB following CaCl_2_-induced arrhythmia (Dianat et al., 2013 ▶). This action is related to increased levels of vitamins C and E as well as glutathione, which scavenge ROS, superoxide anions, and hydroxyl radicals (Priscilla and Prince, 2009 ▶). Also, it was shown that GA through attenuating platlet aggregation has protective actions against arrhythmias (Appeldoorn et al., 2005 ▶). 

Prolonged QT interval is an electerocardiographic manifestation in underlying of cirrhosis (Alqahtani et al., 2008 ▶). Furthermore, it has been indicated that QTc interval prolongation can be associated with enhancement of occurrence of ventricular arrhythmias (De et al., 2003 ▶). Although, in this regard, mechanism is not clear yet, there seems to be a strong correlation between QTc prolongation and cirrhosis representing QTc interval as the important ECG finding in cirrhosis cardiomyopathy (Carey and Douglas, 2005 ▶). It was revealed that GA increased QRS voltage, and reduction of QTc interval in a rat model of liver cirrhosis following induction of bile duct ligation (Badavi et al., 2016 ▶).

**Table 2:** The mechanisms involved in gallic acid effects against cardiovascular diseases

**Study design**	**Effects**	**References**
Isoproterenol-induced cardiotoxicity	↓ECM proteins (collagens type I and III), CTGF, TGF-β1 ↓CPK, CK-MB, and LDH, cTnT ↓HDAC1 or HDAC2 ↓ANP, BNP, βMHC ↓p-JNKs, p-ERK, ↓p-Smad3	(Stanely Mainzen Prince et al., 2009 ▶, Priscilla and Prince, 2009 ▶; Bakheet et al., 2014 ▶; Umadevi et al., 2014 ▶; Ryu et al., 2016 ▶; Jin et al., 2017 ▶)
Doxorubicin‑induced cardiotoxicity	↓CK‑MB, LDH, LDL-c, VLDL, TG,↑HDL ↑Levels of GSH, SOD, CAT ↓MDA activity Elimination of ↑ST segment ↓P wave, QRS complex ↓BP ↓Histopathological changes	(Kulkarni and Swamy, 2015 ▶)
ECG abnormalities	↓ Incidence VT, VF, PVB ↓QT interval prolongation ,↑QRS voltage	(Dianat et al., 2013 ▶; Badavi et al., 2016 ▶)
Cardiac hypertrophy and spontaneously hypertensive	↓Serum levels of CPK, CK-MB, LDH, cTnT ↓Cardiac hypertrophy, infarct size Modulatory effects on LVP, LVEDP, LVSP, LVDP, RPP ↓p-JNKs, p- ERK & GATA6 downregulation	(Umadevi et al., 2014 ▶; piao et al., 2017)
Cardiac ischemia-reperfusion injury	↓CPK, CK-MB, LDH ↑ SOD, CAT, GPx activity & ↓MDA level ↓Hypertrophy, infarct size Preserve the cell membrane and mitochondria & ↓mPTPs opening ↑coronary flow	(Badavi et al., 2014 ▶; Dianat et al., 2014 ▶; Badavi et al., 2017 ▶)
Diabetes- induced myocardial dysfunction	↓CPK, CK-MB, and LDH, cTnT, BS ↑ SOD, CAT, GPx ↑LVP, LVEDP, LVSP, LVDP, RPP ↑ SOD, CAT, GPx, ↓MDA, ↑NO ↓ Hypertrophy, infarct size & ↑vasodilatory response	(Patel and Goyal, 2011 ▶; Ramezani-Aliakbari et al., 2017 ▶)
Immobilization induced stress in myocardium	↑SOD, CAT, GPx activity ↓LPO markers (TBARS, LOOH) Preserved the structural integrity of heart	(Rather and Saravanan 2013 ▶)

**Effect of gallic acid on cardiac ischemia/reperfusion injury**

Ischemia-reperfusion (I/R) injury results in cellular insults in an organ following ischemia which further aggravate during the re-supply of blood circulation (Akbari et al., 2018 ▶). It substantially affects hepatic (Akbari et al., 2017 ▶), renal (Mard et al., 2017 ▶), gastric (Odukanmi et al., 2017 ▶), brain (Sanderson et al., 2013 ▶), and cardiac functions (Hausenloy and Yellon, 2013 ▶). The pathophysiology of ischema-induced cell damage is calcium dyshomeostasis, activation of phospholipases, proteases, and endonucleases and generation of ROS which induce oxidative stress and affect cellular macromolecules (Rhodena et al., 2002 ▶), activating the ROS-dependent processes, opening of mitochondrial permeability transition pore (mPTP) (Collard and Gelman, 2001 ▶), and electron transformation chain (ETC) disruption (Paradies et al., 1999 ▶). 

The overproduction of ROS in the absence of antioxidant system has been regarded as a possible mechanism in the pathogenesis of I/R injury (Arabi et al., 2017 ▶). Although, ROS at physiological concentrations is useful for cardiac molecular signaling pathways, but at the high pathological levels induces inverse effects on this organ. Furthermore, production of ROS, during ischemia and at the early stage of re-oxygenation can have negative impacts on the heart (Popov et al., 2017 ▶). Reperfusion, in the setting of angioplasty and thrombolysis, reestablishes coronary blood supply and decreases infarct size, but initiates damage to cardiomyocyte (i.e. I/R insult) (Chi et al., 2017 ▶).

It was shown GA preserves the cell membrane and mitochondria against ROS-induced damage by preventing mPTP opening (Badavi et al., 2014 ▶). The antioxidant enzymes of SOD, CAT, and GPx have protective effect against I/R**-**induced damage (Akbari et al., 2017 ▶). Furthermore, studies showed that GA through increasing the capacity of endogenous antioxidant system protected the rat isolated heart against this injury (Badavi et al., 2014 ▶; Dianat et al., 2014 ▶).

**Effect of gallic acid on cardiac remodeling**

 Cardiac remodeling was first discovered in regional dilatation and thinning of infarcted myocardium in rats (Hochman and Bulkley, 1982 ▶). This term was attributed to alterations in the cardiac structure such as cardiomyocyte loss, cardiac hypertrophy, and changes of extracellular matrix, metabolic problems, and mitochondrial impairment. Cardiac remodeling leads to cardiac hypertrophy, ﬁbrosis and a progressive impairment in contractility and relaxation in the heart and eventually cardiac failure (Schirone et al., 2017 ▶). 

Cardiac hypertrophy is a manifestation of hypertrophic cardiomyopathy (HCM). Dilated cardiomyopathy (DCM) is a type of HCM and contributes to heart fibrosis (Inagaki et al., 2008 ▶). It is manifested by enhancement of cardiac mass, and protein synthesis rate, sarcomeric reorganization, and activation of atrial natriuretic peptide, brain natriuretic peptide, β-myosin heavy chain, and skeletal α-actin. It is usually associated with fibrosis which is determined by aggregation of collagen type I and fibronectin (Jeong et al., 2005 ▶). Multiple hypertrophic parameters initiate several signaling pathways by stimulating downstream targets. One of the most important pathways is the mitogen-activated protein kinase (MAPK) (Rose et al., 2010 ▶) that divides into p38 kinases, c-Jun N-terminal kinase (JNK) and extracellular signal regulated kinase (ERK). It has been reported that these kinases decreased by GA pretreatment (Ryu et al., 2016 ▶). Furthermore, GA prevents β-adrenergic agonist-induced cardiac hypertrophy (Ryu et al., 2016 ▶). GA also decreased the increased heart weight to body weight ratio in streptozotocin-induced diabetic rats (Patel and Goyal, 2011 ▶).

 GATA6 is a major hypertrophic regulator and its overexpression induces cardiac hypertrophy both in vivo and in vitro (Liang et al., 2001 ▶). It was revealed that attenuation of myocardial hypertrophy by GA accompany with GATA6 downregulation. GA also downregulates cardiac Nox2 expression and Nox2-induced oxidative stress response via suppression of GATA6 or by affecting DNA-binding activity of GATA6 (Jin et al., 2017 ▶).

 The term cardiac ﬁbrosis is attributed to aggregation of types I and III collagen, and extracellular matrix (ECM) crosslinking which lead to stiffening of the cardiac chamber, and disturbances in cardiac elasticity and diastolic function (Segura et al., 2014 ▶). Taken together, the progression of ﬁbrosis requires increased synthesis of matrix metalloproteinases (MMPs) (Kandalam et al., 2011 ▶), activation of proﬁbrotic mediators, such as TGF-β, α-smooth muscle actin (α-SMA), platelet-derived growth factor (PDGF), cytokines (Kong et al., 2014 ▶), and differentiation of ﬁbroblasts into myoﬁbroblasts (Wang et al., 2003 ▶). 

It was shown that GA effectively attenuated the cardiac fibrosis in the pre-established HTN mice. This effect may be related to downregulation of ECM proteins collagens of type I and III, and connective tissue growth factor (CTGF). Furthermore, GA decreased the NG- nitro- L -arginine methyl ester (L-NAME) induced transforming growth factor beta 1 (TGF-β1) which is a cytokine and mediator in fibroblast proliferation and ECM accumulation (Pohlers et al., 2009 ▶).

TGF-β1- small mother against decapentaplegic 3 (Smad3) is another signaling network which contributes to fibrosis formation. Smad2 and Smad3 are downstream mediators of TGF-β. In cytoplasm, Smad2 and Smad3 are phosphorylated by activated type I receptors, then they bind Smad4. This complex is then transfered to the nucleus. Smad2 protects fibrosis (Meng et al., 2010 ▶), whereas Smad3 activates it (Qin et al., 2011 ▶). The correlation between MAPK and TGF-β1-Smad3 axis has been reported. GA conserves cardiac hypertrophy and fibrosis via regulation of the MAPK signaling pathway and Smad3-mediated collagen type I expression (Ryu et al., 2016 ▶).

Histone acetyltransferases (HATs) are a category of enzymes that acetylate histones. An imbalance between HATs and histone deacetylases (HDACs) can lead to pathological disorders. Furthermore, alterations in HDAC amounts and enzyme activity are implicated in a variety of diseases, including cardiac hypertrophy, and fibrosis. It was shown that antihypertrophic or antifibrosis effect of GA is mediated by the regulation of HDAC1 or HDAC2 (Jin et al., 2017 ▶). 

**Effect of gallic acid on drugs-induced cardiotoxicity**

Cardiotoxicity is a well-known complication of multiple drugs which (Meinardi et al., 2000 ▶) includes a broad range of cardiac characteristics from little alterations in several parameters such as blood pressure (BP) and arrhythmias to cardiomyopathy (Arola et al., 2000 ▶). Doxorubicin (DOX)-induced cardiomyopathy is related with an elevation oxidative stress in the heart which is manifested by lipid peroxidation, decreased antioxidants capacity and sulfhydryl groups levels (Octavia et al., 2012 ▶).

 DOX is one of the most important anti-neoplastic agents to treat a number of diseases; however, its use is limited owing to the dose‑dependent cardiotoxicity which leads to dilated cardiomyopathy with fetal congestive heart failure (Mohan et al. 2006 ▶). Electrocardiograph (ECG) changes are the major findings for diagnosis of cardiac damage. The ECG findings following DOX toxicity are elevation of ST segment, reduction of P wave, QRS complex, and prolongation of QT interval (Octavia et al., 2012 ▶). 

 Pretreatment with GA eliminated DOX‑induced altered ECG, and reduced serum concentrations of CK‑MB, LDH, low‑density lipoprotein-cholesterol (LDL-c), and serum triglycerides. This effect may be related to preservation of the normal structure of cardiac myocytes due to restoring the antioxidants of GSH, SOD, and CAT, and hence decreasing the entrance of mentioned parameters to the blood circulation. The lipidlowering action of GA is attributed to suppression of hepatic cholesterol biosynthesis, increased fecal bile secretion and activation of receptor-mediated catabolism of LDL**-**c, and decreases in serum levels of triglycerides, LDL**-**c, very low‑density lipoprotein‑cholesterol through inhibiting lipogenesis (Kulkarni and Swamy, 2015 ▶). 

 Among catecholamines, isoproterenol (ISP), is a well-known drug for induction of MI in animal models for investigation of the effects of drugs on cardiac function (Stanely Mainzen Prince et al., 2009 ▶). It has been shown that in mice, infusion of ISP (up to 2 weeks) leads to cardiac hypertrophy which is manifested by enhancement of wall thickness of left ventricle (LV) (Ryu et al., 2016 ▶). Mechanisms suggested for explaining ISP-induced cardiac insult are production of abundant cytotoxic free radicals and increased activities of lysosomal enzymes (Stanely Mainzen Prince et al., 2009 ▶).

Excessive production of free radicals may result in the loss of function and integrity of myocardial membranes (Priscilla and Prince, 2009). GA preserves the integrity of lysosomal membrane by maintaining the activities of lysosomal enzymes in the serum and heart due to antilipoperoxidative and antioxidant impacts of its (Stanely Mainzen Prince et al., 2009 ▶; Umadevi et al., 2012 ▶). In addition, it was reported that GA decreased the ISP-induced phosphorylation JNK1/2 and ERK1/2 in vivo (Ryu et al., 2016 ▶). Furthermore, it was presented that administration of GA suppressed collagen aggregation due to ISP in mice and it attenuated the mRNA and protein expression of fibronectin, collagen type I, and α-smooth muscle actin (Ryu et al., 2016 ▶).

**Effect of gallic acid on DM-induced CVDs**

 Diabetic mellitus (DM) is a metabolic problem which is associated with high blood glucose owing to disturbance in insulin secretion, insulin impact, or both (Olusoji et al., 2017 ▶) which accompany CVDs (Perazzoli et al., 2017 ▶). NO, prostacyclin and endothelium-derived hyperpolarization are major signaling pathways contributing to relaxation of endothelium (Ng et al., 2017 ▶).

It was reported that chronic DM affects endothelial function, but not vascular smooth muscle which is associated with enhancement of oxidative stress (Pannirselvam et al., 2005 ▶). 

It was shown that endothelium-dependent vasodilatory response of the mesenteric vascular bed (MVB) to histamine, is dramatically attenuated and it is related to both NO and prostaglandin pathways (Pannirselvam et al., 2005 ▶).

The incidence of myocardial dysfunction is two to five times higher in diabetic patients than nondiabetics. Hyperglycemia, hyperlipidemia, oxidative stress, and production of advanced glycation end products have all been contributed to the pathogenesis of diabetes (Patel and Goyal, 2011 ▶). It was shown that DM increases oxidative stress and reduces antioxidant capacity in I/R hearts (Suwalsky et al., 2016 ▶). 

GA increased the decreased hemodynamic parameters such as left ventricular pressure (LVP), left ventricular end diastolic pressure (LVEDP), left ventricular end diastolic and systolic pressure (LVEDP, and LVSP) and Left ventricular developed pressure (LVDP), rate pressure product (RPP), and antioxidant capacity of SOD, CAT, and GPx as well as NO. Indeed, this agent decreased infarct size, and cardiac marker enzymes including CPK, CK-MB, and LDH, and cTnT following I/R injury in hearts isolated from rats with alloxan-induced DM. Other elevated parameters such as blood sugar (BS), and MDA level also decreased in this experimental model (Ramezani-Aliakbari et al., 2017 ▶).

## Discussion

CVDs including coronary artery disease, hypertension, arrhythmia, heart failure, myocardial infarction, cardiac remodeling, and cardiotoxicity are the major cause of mortality and have become one of the most important health challenges in developing countries. The pathophysiology of CVDs are overproduction of toxic lipid metabolites, elevation of cardiac marker enzymes, hemodynamic parameters disturbances, histopathological alterations and decresing of antioxidant activity. In ethno-pharmacology science, study on natural herbal products with different mechanisms is aimed for progress of new medication because chemical drugs have various disadvantages.

 GA is one of the most important herbal products which has beneﬁcial effects on CVDs. GA has protective effects on drugs-induced cardiotoxicity through decreasing the ECM proteins, CTGF, TGF-β1 CPK, CK-MB, cTnT, LDH, LDL-c, VLDL, TG, HDAC1 or HDAC2, ANP, BNP, βMHC, p-JNKs, p-ERK, p-Smad3, MDA levels, and histopathological alterations and increasing levels of HDL, GSH, SOD, and CAT. Furthermore, GA attenuates ECG abnormalities via decreasing incidence of VT, VF, PVB, QT interval prolongation, and elevation of QRS voltage.

The effect of GA on cardiac ischemia-reperfusion injury attributed to mitigating levels of CPK, CK-MB, LDH, MDA, infarct size, mPTPs opening and preservation of cell membrane, enhacement of SOD, CAT, GPx activity and coronary blood flow. In addition, GA has regulatory action on cardiac hypertrophy and hypertension through reducing serum levels of CPK, CK-MB, LDH, cTnT, cardiac hypertrophy, infarct size, and down-regulating of p-JNKs, p- ERK, GATA6 and modulating of hemodynamic parameters including LVP, LVEDP, LVSP, LVDP, RPP. On the other hand, protective action of GA on diabetes- induced myocardial dysfunction attributed to attenuating of serum levels of CPK, CK-MB, and LDH, cTnT, BS, and decreasing MDA levels, myocardial hypertrophy, infarct size and increasing antioxidant activity of SOD, CAT, GPx, and hemodynamic parameters such as LVP, LVEDP, LVSP, LVDP, RPP and vasodilatory response. GA via increasing SOD, CAT, and GPx activities, decreasing LPO markers, and preserving of the structural integrity of heart tissues also reduces immobilization induced stress in myocardium. 

GA also showed protective role against vascular diseases through molecular mechanisms of increasing NO level, down-regulation of protein expression of Nox2, angiotensin II, angiotensin II-converting enzyme, CaMKIIδ, and apoptotic genes including BAX, and p53 mRNA level, as well as decreasing of vascular calcification through BMP2–smad1/5/8 signaling pathway inhibition. 
